# Mortality Prediction in Sepsis With an Immune-Related Transcriptomics Signature: A Multi-Cohort Analysis

**DOI:** 10.3389/fmed.2022.930043

**Published:** 2022-06-30

**Authors:** Louis Kreitmann, Maxime Bodinier, Aurore Fleurie, Katia Imhoff, Marie-Angelique Cazalis, Estelle Peronnet, Elisabeth Cerrato, Claire Tardiveau, Filippo Conti, Jean-François Llitjos, Julien Textoris, Guillaume Monneret, Sophie Blein, Karen Brengel-Pesce

**Affiliations:** ^1^EA 7426 “Pathophysiology of Injury-Induced Immunosuppression”, Joint Research Unit Université Claude Bernard Lyon 1 – Hospices Civils de Lyon – bioMérieux, Lyon, France; ^2^Open Innovation and Partnerships (OIP), bioMérieux S.A., Marcy-l’Étoile, France; ^3^Data Science, bioMérieux S.A., Marcy-l’Etoile, France; ^4^ Immunology Laboratory, Edouard Herriot Hospital – Hospices Civils de Lyon, Lyon, France; ^5^Anaesthesia and Critical Care Medicine Department, Hospices Civils de Lyon, Edouard Herriot Hospital, Lyon, France; ^6^Medical Affairs, bioMérieux S.A., Marcy-l’Etoile, France

**Keywords:** sepsis, transcriptomics, predictive modeling, gene expression analysis, mortality, biomarker discovery

## Abstract

**Background:**

Novel biomarkers are needed to progress toward individualized patient care in sepsis. The immune profiling panel (IPP) prototype has been designed as a fully-automated multiplex tool measuring expression levels of 26 genes in sepsis patients to explore immune functions, determine sepsis endotypes and guide personalized clinical management. The performance of the IPP gene set to predict 30-day mortality has not been extensively characterized in heterogeneous cohorts of sepsis patients.

**Methods:**

Publicly available microarray data of sepsis patients with widely variable demographics, clinical characteristics and ethnical background were co-normalized, and the performance of the IPP gene set to predict 30-day mortality was assessed using a combination of machine learning algorithms.

**Results:**

We collected data from 1,801 arrays sampled on sepsis patients and 598 sampled on controls in 17 studies. When gene expression was assayed at day 1 following admission (1,437 arrays sampled on sepsis patients, of whom 1,161 were alive and 276 (19.2%) were dead at day 30), the IPP gene set showed good performance to predict 30-day mortality, with an area under the receiving operating characteristics curve (AUROC) of 0.710 (CI 0.652–0.768). Importantly, there was no statistically significant improvement in predictive performance when training the same models with all genes common to the 17 microarray studies (*n* = 7,122 genes), with an AUROC = 0.755 (CI 0.697–0.813, *p* = 0.286). In patients with gene expression data sampled at day 3 following admission or later, the IPP gene set had higher performance, with an AUROC = 0.804 (CI 0.643–0.964), while the total gene pool had an AUROC = 0.787 (CI 0.610–0.965, *p* = 0.811).

**Conclusion:**

Using pooled publicly-available gene expression data from multiple cohorts, we showed that the IPP gene set, an immune-related transcriptomics signature conveys relevant information to predict 30-day mortality when sampled at day 1 following admission. Our data also suggests that higher predictive performance could be obtained when assaying gene expression at later time points during the course of sepsis. Prospective studies are needed to confirm these findings using the IPP gene set on its dedicated measurement platform.

## Introduction

Sepsis – a dysregulated immune response to severe infection leading to acute organ dysfunction (1) – is the third leading cause of death worldwide and the main cause of in-hospital mortality (2, 3). Despite more than 100 randomized clinical trials attempting to manipulate the host response to improve sepsis outcomes, sepsis care remains mainly supportive, limited to hemodynamic support, early antibiotic treatment and source control (4). In contrast to what is seen in the treatment of cancer, the aim of delivering precision medicine in sepsis remains far from attained: new tools and strategies are urgently needed to progress toward individualized patient care in sepsis (5, 6).

*Why have all clinical trials in sepsis failed?* (7). One reason is that they have not taken into account the significant heterogeneity in the epidemiology, microbiology and immunology of this syndrome. The immune response in sepsis is highly complex and dynamic, involving both pro- and anti-inflammatory mechanisms, with substantial intra- and inter-individual variability (8, 9). While its initial phase is characterized by uncontrolled inflammation responsible for tissue injury, sepsis patients also display markers of a profound immunosuppression (10), linked to a high prevalence of secondary opportunistic infections (11, 12) and contributing to significant mortality in sepsis survivors (13). Thus, trials are investigating whether immune-suppressing therapies such as interleukine (IL) 1 receptor antagonist (IL-1Ra) and anti–IL-6 could dampen the early cytokine storm, and conversely whether immune-stimulatory agents such as IL-7, granulocyte macrophage-colony stimulating factor (GM-CSF), and interferon gamma (IFN-γ) could reverse sepsis-induced immunosuppression (14).

To identify sub-groups of patients with reduced heterogeneity and a higher likelihood to respond favorably to such targeted therapies, it is crucial to use appropriate biomarkers (15, 16). For example, a low expression of human leukocyte antigen–DR on monocytes (mHLA-DR) can be used as a surrogate marker for monocyte anergy and decreased antigen presentation (17), and has been used as an inclusion criterion in the GM-CSF trial (18). However, its dissemination at the point-of-care has been limited, mainly because its accurate measurement is time-consuming and requires dedicated specialized personnel and equipment, and also because – as a univariate biomarker – it may fail to capture the global complexity of sepsis immunology.

More recent biotechnological and analytical advances have prompted the use of -omics technologies - mostly transcriptomics - to probe the immune response in sepsis, hoping that this approach could uncover important mechanisms of immune regulation and help identify biomarkers to inform targeted therapeutic strategies in sepsis (16, 19). By assaying messenger RNA (mRNA) transcripts in peripheral blood leukocytes and using unsupervised machine learning (ML) methods, sub-groups of sepsis patients whose distinct patterns of gene expression (GE) can be linked to distinct immune states, so-called « endotypes », have been identified. For instance, the Dutch Molecular Diagnosis and Risk Stratification of Sepsis (MARS) project identified four distinct sepsis endotypes named MARS 1 to 4, with patients in the MARS 1 cluster showing a pronounced decrease in expression of genes corresponding to key innate and adaptive immune cell functions and a decreased 28-day survival (20); and the United Kingdom Genomic Advances in Sepsis (GAinS) study identified two distinct sepsis response signatures named SRS 1 and SRS 2, with SRS 1 patients having an immunosuppressed status and higher 14-day mortality (21).

Importantly, there is only partial overlap in differentially expressed genes of the MARS 1 and SRS 1 clusters, raising the question of the generalizability of these signatures. This could be explained by the limited sample size of both studies; the redundancy in the information carried by multiples genes belonging to common biological pathways; and the sampling of patients from restricted ethnic backgrounds and geographic areas. In order to increase the potential to generalize transcriptomics studies in sepsis, one strategy is to leverage biological and technical heterogeneity across a large number of studies taken from diverse clinical backgrounds and profiled using different platforms (22). To this end, Stanford-based investigators have collected publicly available GE data sets sampled from sepsis patients, implemented a modified type of array normalization that uses the ComBat empirical Bayes normalization method (an algorithm called COCONUT, for COmbat CO-normalization Using coNTrols) and used a supervised learning approach to identify a gene signature predictive of 30-day sepsis mortality (23).

However, while this approach has focused on finding a gene signature with the broadest generalizability across populations and the highest predictive performance, it does not provide a mechanistic insight into the pathways involved in disease trajectories. Further, none of the above-mentioned signatures have incorporated prior knowledge on immunological abnormalities in sepsis, nor was devised precisely to discriminate between sub-groups of sepsis patients that could be targeted by specific immunomodulatory agents. Finally, they were devised using microarray data, while a point-of-care device targeting these gene sets would most likely use another technology to measure gene expression, raising the question of the transferability across platforms.

To circumvent these obstacles, we are working on an Immune Profiling Panel (IPP) prototype, a multiplexed transcriptomic assay that uses the FilmArray technology to quantify mRNA expression in whole blood and deliver results in less than an hour (24, 25). This prototype test, which has not been submitted for regulatory review at the time of this writing, may someday be able to provide clinicians with timely information about the immune system of sepsis patients and potentially aid in providing appropriate care. Selection of the IPP gene set was based on existing knowledge on genes related to relevant outcomes in sepsis (mortality prediction, sepsis-associated immunosuppression, susceptibility to secondary infections); technical performance of the selected targets in multiplex quantitative polymerase chain reaction (qPCR); and the goal to attain a balanced representation of pathways involved in sepsis immunopathology (such as monocyte anergy, antigen presentation, lymphocyte exhaustion, etc.) (26–29).

However, the performance of the whole IPP gene set to predict 30-day mortality in sepsis has not been evaluated in a large heterogeneous cohort of sepsis patients. To this end, we decided to: 1) collect publicly available microarray data sets of sepsis patients with patient-level information on mortality; (2) co-normalize data sets using COCONUT; (3) optimize ML models using the expression of the IPP gene set on day 1 following admission as input and 30-day mortality as the outcome of interest to evaluate the predictive performance of the IPP signature. Additional objectives were to evaluate if better predictive performances could be attained either by using another gene signature, or by using GE data sampled more than 2 days after hospital admission.

## Methods

### Data Collection and Pre-processing

We searched NCBI GEO and EMBL-EBI ArrayExpress databases for studies with the following inclusion criteria: (1) publicly available GE data from micro-array experiments collected by whole blood sampling, with at least one sample collected at day 1 following hospital or intensive care unit (ICU) admission; (2) adult or pediatric patients with sepsis, according to Sepsis-1 (30), Sepsis-2 (31) or Sepsis-3 (32) definitions; (3) individual patient data on mortality (assessed between 28 and 30 days after blood sampling); (4) at least 5 control patients (healthy volunteers or patients with non-septic inflammation), which was mandatory for co-normalization across studies. Data sets using endotoxin or lipopolysaccharide infusion as a model for inflammation or sepsis, as well as datasets derived from sorted cells and RNAseq experiments were excluded.

We collected normalized GE data from selected studies when it was available, and inspected normalization visually by plotting individual patient data for each study. In case normalized data was not available, raw GE data was downloaded and normalized using the gcRMA method (R package affy) for *Affymetrix* chips, and normal-exponential background corrected and quantile normalized (R package limma) for *Agilent* and *Illumina* chips. When several microarray probe sets pointed toward one common gene under the HUGO gene nomenclature data base, we used the collapseRows function in the R package WGCNA to select the probe set with the highest mean value (MaxMean method) (33).

Individual patient data related to demographics and clinical characteristics were also extracted when available, including data on age, gender, ethnicity, clinical severity scores, and bacterial vs. viral origin of sepsis.

### Co-normalization Using COCONUT

Comparison of GE data from different microarray studies is limited by different background measurements for each gene between microarrays, and potential batch effects among studies using the same types of microarrays. To analyze pooled data from different studies, co-normalization methods must be applied in such a way that: (1) no bias is introduced that could influence final classification; (2) there should be no change in the distribution of a gene within a study; and (3) a gene should show the same range of distributions between studies after normalization (34). To this end, we used the R package COCONUT (35), which implements a modified version of the ComBat empirical Bayes normalization method (36), using the assumption that all healthy/control patients from different studies come from the same distribution. All cohorts are split into healthy/control and diseased (sepsis) patients; the healthy components undergo parametric ComBat co-normalization without covariates; the ComBat estimated parameters are obtained for each data set for the healthy/control component and then applied to the diseased component.

### Model Selection, Performance Metrics, Hyperparameter Tuning

Prior to model training, we randomly split the ComBat-corrected GE data into a discovery data set (70%) and a validation data set (30%). The discovery set was used to train several classification algorithms, taking GE data related to the IPP genes as input and 30-day mortality as outcome: logistic regression with L1 (lasso), L2 (ridge) and mixed (elastic net) regularization, random forest, support vector machines with linear and radial kernels and partial least squares-discriminant analysis. Mortality was considered as a binary variable because time-to-event data were not available in most public data sets.

Hyperparameter optimization was performed to select models with the highest mean area under the receiver operating characteristic (ROC) curve (AUROC) using 5 repetitions of 10-fold cross-validation. Alternatively, the area under the precision recall curve (AUPRC) was used as a performance scoring metric because our discovery data set had an imbalanced distribution of the outcome (with ∼19% mortality) (37). Furthermore, to mitigate the negative impact of data imbalance on model training, we used several oversampling strategies, including the Synthetic Minority Oversampling Technique (SMOTE) on the discovery data set prior to hyperparameter tuning (38).

For each optimized model, we evaluated performance by computing the AUROC and its confidence interval (DeLong method) on the validation set.

### Models and Feature Sets Comparisons

The IPP gene set contains 26 immune-related genes and 3 genes used for normalization, and we used those the total of 29 as input in the IPP models (Supplementary Table 1). To compare the predictive performance of the IPP gene set to that of the best possible signature derived from the pooled data set assembled from publicly available microarray data, we trained the same machine learning (ML) models, taking all genes common to all included studies as input (“all genes” models, *n* = 7,122 genes). To see if improvement in predictive performance from the IPP gene set to the total gene pool was due to the fact that the IPP gene set did not contain the best set of predictors, or solely a consequence of it having a limited number of predictors, we selected the 29 genes with the highest feature importance in the best performing “total gene pool” model and re-ran ML models using the “top 29 genes” set as input. Finally, we compared the IPP gene set to the “all genes” and “top 29 genes” sets by comparing ROC curves obtained by prediction on the validation set.

To determine if gene expression data could yield different predictive information on mortality if mRNA is sampled at time points beyond patient admission, we trained models on 2 data sets: (1) the “day 1” data set was a subset of the whole co-normalized data set, restricted to cohorts with available GE data for all the IPP genes, sampled at day 1 following enrolment; (2) the “day > 2” data set was a subset of the whole co-normalized data set, restricted to cohorts with available GE data for all the IPP genes, sampled at time points 3 to 7 days following enrolment. Each of these 2 data sets were split in discovery and validation sets as described above.

Finally, we sought to assess how IPP could be used as a tool for prognostication at the patient level. We used IPP models and found optimal thresholds of sensitivity and specificity using the top-left method on the “day 1” and “day > 2” data sets, enabling us to define 2 groups based on the predicted probability of death (low- and high-risk groups). Finally we computed and compared observed 30-day mortality rates in the low- and high-risk groups using appropriate statistical tests (see below).

### Statistical Analysis and Software

To compare demographics and clinical features in the discovery and validation data sets, we used the Wilcoxon rank sum test. To compare predictive performance between models, we compared ROC curves computed using the same test set with DeLong’s test for correlated data. To compare proportions of dead patients between different risk groups obtained with IPP genes, we used the chi-squared test or Fisher’s exact test, as appropriate. Significance levels for *p*-values were set at 0.05 and analyses were two-tailed. Statistical analyses were performed using R (v3.6.2) with packages from the BioConductor library, the tidyverse collection, caret and COCONUT, as well as on Python 3 with the scikit-learn machine learning library.

## Results

### Studies Included in the Analysis, Discovery and Validation Sets

Twenty studies fulfilled our inclusion criteria (20, 39–58). Of these, three studies (40, 48, 56) did not contain data on three genes included in the IPP gene set (TDRD9, CD274 and ARL14EP) because associated probes were not on the chip used in these studies (Affymetrix Human Genome U133A 2.0 Array), and were subsequently removed from analysis.

The remaining 17 studies included 2,399 arrays, with 1,801 arrays from sepsis patients and 598 arrays from controls (Table 1). The “day 1” data set included 1,437 arrays sampled on sepsis patients at day 1, of whom 1,161 were alive and 276 (19.2%) were deceased at day 30 following enrolment. As presented in Table 2, demographics and clinical characteristics were similar in the discovery (*n* = 1,007) and validation (*n* = 430) sets obtained after random splitting of the “day 1” data set.

**TABLE 1:** Characteristics of the cohorts, patients and microarray data included in the study.

Dataset accession	First author	Country	CA vs. HCA**	Time points	Age	Sex (%males)	Arrays	Patients	Controls	Sepsis	Bacterial	Viral	Alive	Deceased	Chip	Normalization method
GSE27131	Berdal	Norway	CA	d1 d6 d7	41.1	85.7	21	14	7	7	0	7	5	2	Affymetrix	RMA
GSE32707	Dolinay	United States	CA	d1	57.1	54.2	103	103	55	48	NA	NA	86	17	Illumina	Quantile
GSE40586	Lill	Estonia	CA	d1	46.1	NA	39	39	18	21	21	0	19	2	Affymetrix	RMA
GSE66099	Wong	United States	CA	d1	3.7	63.1	276	276	77	199	NA	NA	248	28	Affymetrix	gcRMA
GSE21802	Bermejo-Martin	Canada	CA	d1	NA	NA	15	15	4	11	0	11	7	4	Illumina	Quantile
GSE54514	Parnell	Australia	CA	d1-d5	59.8	41.7	163	54	18	36	36	0	26	10	Illumina	Quantile
GSE20346	Parnell	Australia	CA	d1-d7	NA	NA	55	22	18	4	0	4	22	0	Illumina	Cubic spline
GSE40012	Parnell	Australia	CA	d1-d5	NA	45.5	129	42	31	11	3	11	42	0	Illumina	Quantile
GSE57065	Cazalis	France	CA HCA	d1 d2 d3	62.7	67.9	107	53	25	28	28	0	22	6	Affymetrix	RMA
GSE60244	Suarez	United States	CA	d1	62.1	41.5	158	158	40	118	47	96	158	0	Illumina	Quantile*
GSE65682	Scicluna	Netherland	CA HCA	d1	61	56.8	521	521	42	479	NA	NA	365	114	Affymetrix	RMA + quantile
GSE95233	Tabone	France	CA	d1 d2 d3	62.1	64.7	124	71	20	51	NA	NA	56	17	Illumina	Quantile
E-MEXP-3589	Almansa	Spain	CA	d1	NA	50	16	16	4	12	5	3	16	0	Agilent	Normexp
E-MTAB-1548	Almansa	Spain	HCA	d1	69.2	67.1	155	155	73	82	NA	NA	138	17	Agilent	Normexp
E-MTAB-5273/5274	Burnham	United Kingdom	CA	d1 d3 d5	65.4	53	337	253	10	243	NA	NA	204	39	Illumina	VSN
GSE13015	Planka	Thailand	CA HCA	d1	53.7	54.7	92	92	29	63	63	0	52	20	Illumina	Quantile*
GSE25504	Smith	United Kingdom	CA HCA	d1	0.25	56.8	88	88	44	44	37	5	84	4	Illumina + Affymetrix	Spline

**Total**							**2,399**	**1,972**	**515**	**1,457**	**240**	**137**	**1,181**	**276**		

*Normalization method was not specified in the original study but was verified graphically and assumed to follow the method specified in the table based on usual methods for the associated chip.

**Community- vs. healthcare associated sepsis cases: CA is for community-acquired and HCA for healthcare-associated infections.

**TABLE 2:** Demographics and clinical characteristics in the discovery and validation sets computed with microarray data sampled at day 1 following study enrolment.

	Discovery set (*n* = 1007)	Validation set (*n* = 430)	*P*-value
Age [mean (SD)]	52.09 (26.35)	49.79 (26.83)	0.138
Gender (n,%)			0.142
Female	419 (41.6)	189 (44.0)	
Male	566 (56.2)	225 (52.3)	
NA	22 (2.2)	16 (3.7)	
Infection setting (n,%)			0.133
Community-associated	685 (68.0)	313 (72.8)	
Healthcare-associated	69 (6.9)	30 (7.0)	
NA	253 (25.1)	87 (20.2)	
Microbiology (n,%)			0.972
Viral sepsis	79 (7.8)	33 (7.7)	
bacterial sepsis	143 (14.2)	63 (14.7)	
NA	785 (78.0)	334 (77.7)	
Ethnic background (n,%)			0.976
Asian	46 (4.6)	19 (4.4)	
Black	17 (1.7)	6 (1.4)	
Latino	13 (1.3)	7 (1.6)	
White	52 (5.2)	21 (4.9)	
NA	879 (87.3)	377 (87.7)	
Platform (n,%)			0.708
Affymetrix	552 (54.8)	226 (52.6)	
Agilent	66 (6.6)	28 (6.5)	
Illumina	389 (38.6)	176 (40.9)	
Survival (n,%)	816 (81.0)	349 (81.2)	> 0.999

*NA indicates values missing in the original studies.*

In the 7 studies (43, 45, 46, 51–53, 58) with GE data collected at time points 3 to 7, there were 270 arrays sampled on 173 patients, of whom 134 were alive and 39 (22.5%) deceased at day 30; 122 were used for training and 51 for testing models (Supplementary Tables 2, 3).

**FIGURE 1:**
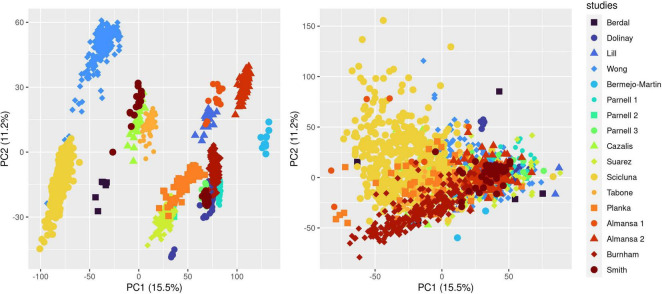
Effect of ComBat co-normalization on patient-level gene expression data assessed by principal component analysis (PCA) across 17 microarray studies. We computed a 2-dimensional PCA plot of individual gene expression data from sepsis patients at day 1 following admission (7,122 genes assessed on 1,437 arrays sampled on 1,437 patients) before (left panel) and after (right panel) ComBat co-normalization using controls with the COCONUT R package. Each of the 17 studies maps to one color, showing how co-normalization attenuates the segregation of individual data points in clusters determined by the study to which they belong.

We ran the COCONUT algorithm on the 17 studies selected for analysis and assessed the effect of co-normalization: (1) on patient-level GE data across studies (Figure 1 and Supplementary Figure 1); (2) at the gene level in controls and cases (Supplementary Figure 2 presenting data for CD3D); (3) for 2 genes in controls and cases, here with CLDN8 (a housekeeping gene, with minimal difference in mean GE between controls and cases and minimal overall GE variance) and CEACAM1, up-regulated during sepsis (Supplementary Figure 3). As expected, visual inspection of these plots confirmed the effect of COCONUT to attenuate the “batch effect” across the selected 17 studies.

### Predictive Performances of the IPP Gene Set at Day 1 Following Admission

**FIGURE 2:**
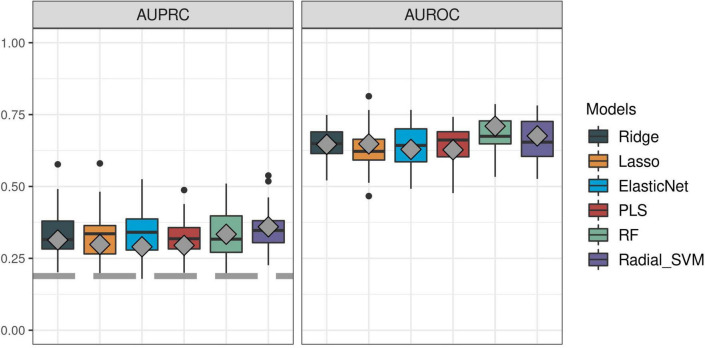
Predictive performance of the IPP gene set on “day 1” discovery and validation sets. We trained machine learning models on the “day 1” discovery (*n* = 1,007) and validation (*n* = 430) data sets by 5 repeats of 10-fold cross-validation, and computed areas under the receiver operating characteristic (AUROC, right panel) and precision-recall curves (AUPRC, left panel) on the resampled discovery set (box plots) and by prediction on the validation set (gray diamonds). Gray dashed line on the AUPRC facet indicates baseline probability of the outcome (death).

**FIGURE 3:**
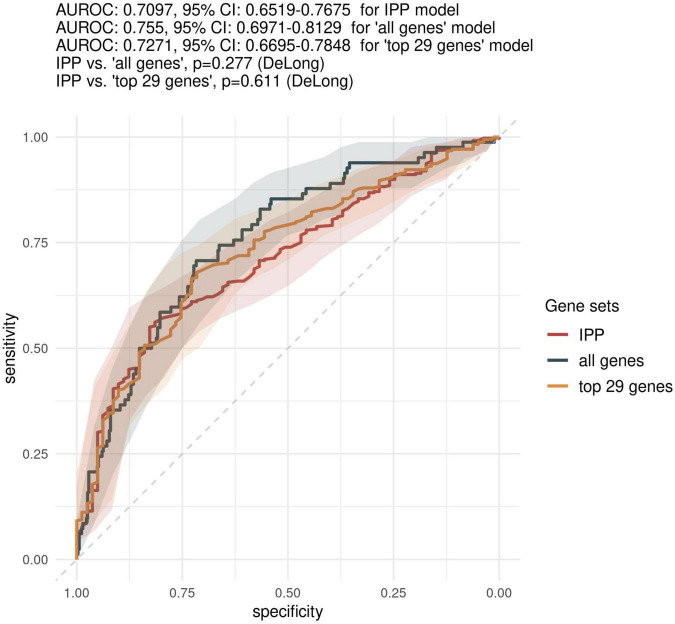
Comparison of predictive performances of the IPP gene set with that obtained with other genes on the “day 1” data set. We compared the predictive performance of the IPP gene set to that obtained with other genes common to the 17 microarray studies by computing ROC curves obtained by prediction on the validation set with IPP, “all genes” and “top 29 genes” models trained on GE data collected at day 1 following admission. Gray areas indicate 95% confidence intervals of corresponding AUROCs.

First, we sought to determine the performance of the IPP gene set to predict 30-day mortality using GE data sampled on the day of patient admission. As shown in Figures 2, 3, the highest predictive performance was obtained by training of a random forest classifier, with an AUROC computed on the validation set of 0.710 (CI 0.652–0.768). Next, to determine if better predictive performance could be extracted from other genes, we ran the same models using all the genes common to the 17 selected studies as input. We found that the highest predictive performance of the “all genes” set (*n* = 7,122 genes) was obtained by training of an L2-penalized logistic regression classifier, with an AUROC computed on the validation set of 0.755 (CI 0.697–0.813), which was not statistically different from the performance obtained with the IPP gene set (*p* = 0.286). In such a logistic regression classifier, it is possible to extract the genes with the highest absolute value of regression coefficients, indicative of the highest predictive performance. Thus, we subsequently trained ML algorithms with the 29 genes with the highest feature importance in the “all genes” model, and obtained an AUROC of 0.727 (CI 0.670–0.785, *p* = 0.610 in comparison to the IPP gene set). In conclusion, we found that the IPP gene set conveyed useful information to predict 30-day mortality with GE data assayed upon patient admission. Furthermore, we found evidence that the predictive power of the IPP gene set was equivalent to the best performing signature extracted from the 17 studies included in our multi-cohort ComBat-normalized data set.

### Predictive Performances at Time Points > Day 2 Following Admission

**FIGURE 4:**
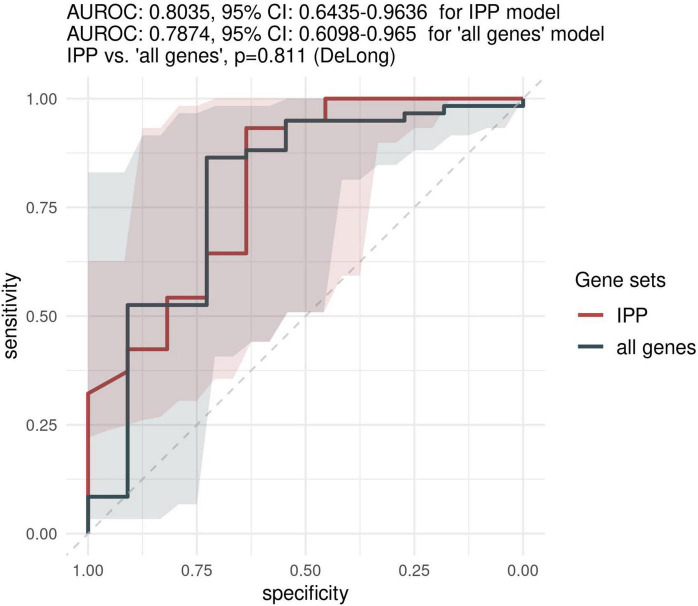
Predictive performance of the IPP gene set on the “days > 2” data set. We assessed the predictive performance of the IPP and “all genes” set by computing ROC curves on the validation set.

Because most of the existing literature on sepsis immunology has shown that more relevant information can be obtained when assessing biomarkers later in the course of disease, we sought to investigate the predictive performance of the IPP gene set when GE data is measured on day 3 following admission or later. As shown in Figure 4, the highest performance of the IPP gene set at days > 2 following admission to predict 30-day mortality was obtained by training of a random forest classifier, with an AUROC computed on the validation set of 0.804 (CI 0.643–0.964). Here again, we found that the IPP gene set yielded similar information to the total gene pool, as we obtained an AUROC on the validation set of 0.787 (CI 0.610–0.965, *p* = 0.811) in the “all genes” best model.

### Interest of IPP for Prognostic Enrichment

The ROC curve provides generic information on the performance of a binary classifier over a range of possible thresholds, but this information might be of limited relevance to clinicians aiming to determine the probability of an event for a specific patient, given the result of the test. To investigate how IPP could be used for prognostic enrichment, we used the optimized ML models obtained with the “day 1” and “days > 2” discovery data sets, computed ROC curves based on predictions on the validation sets, extracted thresholds based on the closest top-left method, and calculated the mortality rates in patients of the validation sets below (low-risk group) and above (high-risk group) this threshold.

**FIGURE 5:**
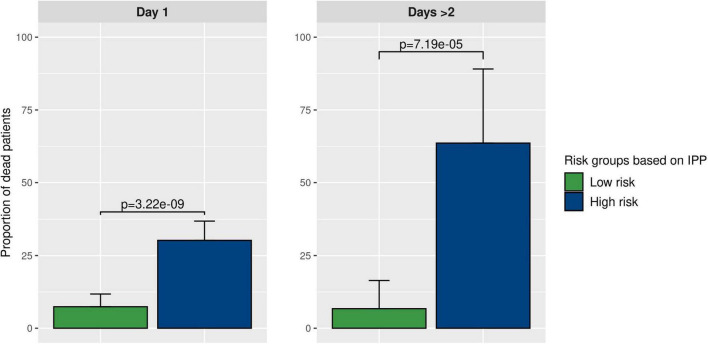
Prognostic enrichment with the IPP tool. We used the best IPP models (trained on the “day 1” and “days > 2” discovery sets) and computed a test threshold using the top-left method on corresponding validation sets. This enabled us to divide the validation sets in 2 sub-groups with a low and a high predicted risk of death. Then, we compared the actual proportion of sepsis patients deceased at day 30 in both sub-groups, to assess if IPP could be used for prognostic enrichment at the bedside.

As shown in Figure 5, using gene expression data from the “day 1” data set, 30.2% (CI 24.2–36.8%) of patients in the high-risk group were dead at day 30, as compared to 7.4% (CI 4.3–11.8%, *p*-value < 10E-8) in the low-risk group. Furthermore, using gene expression data from the “days > 2” data set, we found that 63.6% (CI 30.8–89.1%) of patients in the high-risk group were dead at day 30, compared to 8.5% (CI 2.8–18.7%, *p*-value < 10E-4) in the low-risk group. This indicated that using IPP at the bedside could help clinicians identify a sub-group of patients with higher 30-day mortality early-on during the course of sepsis.

## Discussion

The main finding of our study is that the IPP gene set has good overall performance to predict 30-day mortality, as assessed using microarray data sampled at day 1 following admission in a large and heterogeneous cohort of sepsis patients, with best model showing an AUROC of 0.710 (95% CI 0.652–0.768). IPP was designed using existing knowledge on sepsis immunology and pathophysiology, with the aim to assess the immune system of sepsis patients in a multifaceted manner, and this study demonstrates that the selected immune-related genes also provide predictive information on all-cause mortality. Furthermore, this information can be captured using retrospective and highly heterogeneous data collected on microarrays, even though the IPP tool is based on a PCR assay.

Importantly, predictive performance obtained with all the genes common to all microarrays (>7,000 genes) was not statistically different from that obtained with the IPP genes. It is still possible that the IPP gene set does not capture all the information available in GE data to predict 30-day mortality, but for important technical reasons (e.g., the limited multiplexing capabilities of most commercially available PCR-based assays), models including a large feature set would not be easy to implement at the bedside. This would mandate finding the optimal trade-off between statistical performance and technical constraints to identify the best number of features to include in the assay. Furthermore, models with a high number of predictors are prone to overfitting, which could limit the prognostic accuracy of gene sets across different technological platforms or in different clinical settings. Overall, these results demonstrate that the IPP gene set can capture similar information on 30-day mortality in sepsis as the total gene pool common to 17 microarrays, but with the potential to deliver actionable results in less than an hour, directly at the point of care.

One key aspect of our analysis pipeline is the use of publicly available GE data and batch-effect correction using the ComBat algorithm, which follows a strategy developed by a group from Stanford University (22, 34, 39, 59). Conceptually, pooling together highly heterogeneous data collected in different clinical settings has the potential to increase the generalizability of gene signatures to populations with different ethnic backgrounds and disease phenotypes. However, one can question the relevance of this approach when looking in detail at the wide variability in the demographics and clinical characteristics of the patients included in our multi-cohort analysis. Whether or not there are in fact shared pathophysiological mechanisms and common immunological pathways in children vs. adults, in viral vs. bacterial sepsis, or in ICU vs. ward patients, remains to be fully investigated to demonstrate the usefulness of this strategy.

The IPP prototype has been designed to be run on a dedicated real-time multiplex PCR platform, whereas GE data used in our analysis was collected on microarrays, which raises the question of cross-platform transferability of transcriptomics assays. Given the sometimes weak correlation in expression levels of the same gene target measured on one given sample but different technology, it is highly possible that the real association between our gene signature (as measured with the IPP tool) and 30-day mortality might not be accurately recapitulated in our study. While many gene signatures have been devised for diagnosis and prediction in sepsis, none so far has been proven robust enough to be translated into a clinically usable tool, in part because good statistical performance seen during the conception phase was not reproduced on prospectively collected new patient data, especially if analyzed on a different platform (60). In recent studies for instance, a gene signature devised using microarray data did not show major improvement in predictive power compared to usual severity scores (SAPS 3 and APACHE II) when tested on prospectively collected patient samples processed on the NanoString nCounter platform (60, 61). In line with this, a prospective multicenter study [IMPACCT (62)] is currently enrolling sepsis patients to better evaluate the predictive performance of the IPP gene set when used on its dedicated platform.

Independent of the question of cross-platform transferability, transcriptomics-based diagnostic tools in sepsis might fail to take into account all the relevant information available to predict key outcomes. For instance, there are validated and widely-used clinical severity scores that can predict mortality in intensive care patients with moderate discrimination but wide generalizability and at virtually no added cost. Thus, when evaluating a transcriptomics-based tool, we should verify that GE data provide information independently of the clinical scores. This question was assessed in the Stanford multi-cohort analysis on mortality prediction by running models including both clinical and transcriptomics data, and evaluating the independent effect of GE data on mortality prediction. These analyses showed a consistent (yet not always large) improvement in AUROCs when using genes in addition to clinical data as input (23). Unfortunately, we were not able to run the same analyses, as the majority of publicly available data sets we used did not report patient-level clinical severity data (and because studies that did report data on clinical severity used a wide range of severity scores, limiting their use in our multi-cohort analysis framework). In the same line, it can be argued that for both, clinical and methodological considerations, it would be interesting to include in our prediction models patient-level data on demographics, clinical characteristics and therapeutics (such a steroids, which are known to influence shock severity and sepsis mortality (63), and are also potentially responsible for a change in immune-related GE profile). This argues in favor of prospectively collecting more high quality data on sepsis patients to refine prediction models that would include all relevant information, including clinical and biological, but also genetic, epigenetic, microbiological (etc.), data.

Another inherent limitation of this work is that even though mortality is widely considered an important patient-centered outcome, it is influenced by myriad factors, including many that are not easily modified through medical intervention, which makes it difficult to predict accurately using easily available patient data. Furthermore, it can be argued that even a perfectly calibrated mortality prediction model would fall short of having a positive impact on an individual patient’s care if not coupled with a set of clinical measures meant to improve patient outcomes. In line with this, models designed to predict healthcare-associated infections (HAIs) may be more valuable to clinicians, as they could enable identification of high-risk patients that could be targeted by preventive bundles of cares [e.g., early removal of invasive devices, which are associated with the occurrence of HAIs (12)]. Maybe even more importantly, models designed to identify sepsis endotypes could lead to targeted immune stimulating therapies (10, 64).

Finally, our study suggests that GE data has better performance to predict mortality when mRNA is sampled on day 3 or later following hospital admission. This finding is in line with numerous reports on sepsis biomarkers used to predict mortality or hospital-acquired infections, which consistently show higher performances when biomarkers are assayed after day 3–4 (17, 65). This is also consistent with accumulating data on sepsis immunology, indicating that sepsis-acquired immunosuppression develops in a subset of patients with a worse prognosis only after a few days of acute inflammation (10, 66, 67). Thus, our findings confirm that a transcriptomics tool assessing the host response of sepsis patients to predict mortality could yield more reliable information if assayed at later time points. However, our data must be interpreted with caution, as there were a limited number of patients with GE data available at time points > 2 days, with only 122 patients in the discovery set and 51 (including 11 deaths) in the validation set. In line with this, evaluating if serial measurements of biomarkers can be used to recapitulate disease trajectories in sepsis, and whether this information can be helpful in refining the definition of sepsis endotypes, is the subject of active research (68).

## Conclusion

Through multi-cohort analysis using ComBat co-normalization on microarray data in a heterogeneous group of sepsis patients, we found that the IPP gene set, when assayed at day 1 following hospital admission, can reliably predict all-cause 30-day mortality. Our data also suggest that more information could be extracted from mRNA data if sampled at later time points, when immunological trajectories begin to diverge between sepsis survivors and patients who will eventually die. Since mortality prediction in sepsis is of limited interest to clinicians if not coupled with specific interventions meant to influence disease trajectory and prognosis, using IPP to identify sepsis endotypes or predict HAI is more likely to have a positive impact on the care of patients with sepsis.

## Data Availability Statement

The raw and normalized gene expression data sets analyzed in this study, data on patient demographics, clinical characteristics and outcomes have been deposited publicly and are available from referenced studies and from Synapse (doi: 10.7303/syn5612563). The code written for co-normalization across studies and assessment of predictive performance of gene signatures is available at: https://github.com/lkreitmann-bmx/IPP_mortality_2022.

## Ethics Statement

Ethical review and approval was not required for the study on human participants in accordance with the local legislation and institutional requirements. Written informed consent to participate in this study was provided by the participants’ legal guardian/next of kin.

## Author Contributions

SB, KB-P, and LK: study conception and design. LK, MB, KI, and SB: statistical analysis. M-AC, EP, and EC: data curation. LK: manuscript drafting. AF, CT, FC, J-FL, JT, and GM: critical revision. All authors contributed to the article and approved the submitted version.

## Conflict of Interest

The IPP gene set has been filed for patent protection. LK was employed by, and has received research funding by bioMérieux. MB, AF, KI, M-AC, EP, EC, CT, J-FL, JT, SB, and KB-P were employed by bioMérieux. The remaining authors declare that the research was conducted in the absence of any commercial or financial relationships that could be construed as a potential conflict of interest.

## Publisher’s Note

All claims expressed in this article are solely those of the authors and do not necessarily represent those of their affiliated organizations, or those of the publisher, the editors and the reviewers. Any product that may be evaluated in this article, or claim that may be made by its manufacturer, is not guaranteed or endorsed by the publisher.

## Abbreviations

AUPRC: area under the precision recall curve
AUROC: area under the receiving operating characteristics curve
COCONUT: COmbat CO-normalization Using coNTrols
GE: gene expression
GM-CSF: granulocyte macrophage-colony stimulating factor
IFN- γ: interferon gamma
IL: interleukin
IPP: immune profiling panel
mHLA-DR: human leukocyte antigen–DR
ML: machine learning
PCR: polymerase chain reaction.
