# Supplementary material for: Mortality Prediction in Sepsis With an Immune-Related Transcriptomics Signature: A Multi-Cohort Analysis
Source: Front Med (Lausanne). 2022 Jun 30;9:930043. doi: 10.3389/fmed.2022.930043 (PMC9280291; doi:10.3389/fmed.2022.930043)

Mortality prediction in sepsis with an immune-related transcriptomics signature: a multi-cohort analysis

Supplementary material

Supplementary tables

Supplementary table 1. IPP gene set

Gene	Name
ADGRE3	Adhesion G protein-coupled receptor E3
ARL14EP	Ribosylation factor like GTPase 14 effector protein
BPGM	Biphosphoglycerate mutase
C3AR1	Complement C3a receptor 1
CCNB1IP1	Cyclin B1 interacting protein 1
CD177	CD177 molecule
CD274	CD274 molecule
CD3D	CD3d molecule
CD74	CD74 molecule
CIITA	Class II major histocompatibility complex transactivator
CTLA4	Cytotoxic T-lymphocyte associated protein 4
CX3CR1	C-X3-C motif chemokine receptor 1
GNLY	Granulysin
IFNg	Interferon gamma
IL10	Interleukin 10
IL1R2	Interleukin 1 receptor 2
IL1RN	Interleukin 1 receptor antagonist
IL7R	Interleukin 7 receptor
IP10/CXCL10	Interferon gamma induced protein 10
MDC1	Mediator of DNA damage checkpoint 1
OAS2	2'-5'-oligoadenylate synthetase 2
S100A9	S100 calcium binding protein A9
TAP2	Transporter 2, ATP binding cassette subfamily B member
TDRD9	Tudor domain containing 9
TNF	Tumor necrosis factor
ZAP70	Zeta chain of T cell receptor associated protein kinase 70

Supplementary table 2. Number of arrays per time points in cases and controls

Time point (day)	1	2	3	4	5	6	7	total
**Sepsis patients**	1437	94	159	34	70	7	0	1801
**Control patients**	516	12	8	6	38	0	18	598

Supplementary table 3. Demographics and clinical characteristics in the discovery and validation sets computed with microarray data sampled at day >2 following study enrolment

	Discovery set (n=122)	Validation set (n=51)	p-value
**Age (mean (SD))**	61.88 (15.72)	62.65 (16.32)	0.782
**Gender (%)**			0.226
female	59 (48.4)	18 (35.3)	
male	61 (50.0)	31 (60.8)	
NA	2 (1.6)	2 (3.9)	
**Infection setting = healtcare-associated (%)**	10 (8.2)	2 (3.9)	0.496
**Microbiology (%)**			0.974
viral sepsis	13 (10.7)	6 (11.8)	
bacterial sepsis	42 (34.4)	17 (33.3)	
NA	67 (54.9)	28 (54.9)	
**Platform = Illumina (%)**	99 (81.1)	41 (80.4)	>0.999
**Survival (%)**	94 (77.0)	40 (78.4)	>0.999

NA indicates values missing in the original studies.

<u>Supplementary figures</u>

Supplementary figure 1. Effect of ComBat co-normalization on patient-level gene expression data across the 17 microarray studies
We computed violin plot of total gene expression in each of the 17 studies before and after ComBat co-normalization using controls with the COCONUT.

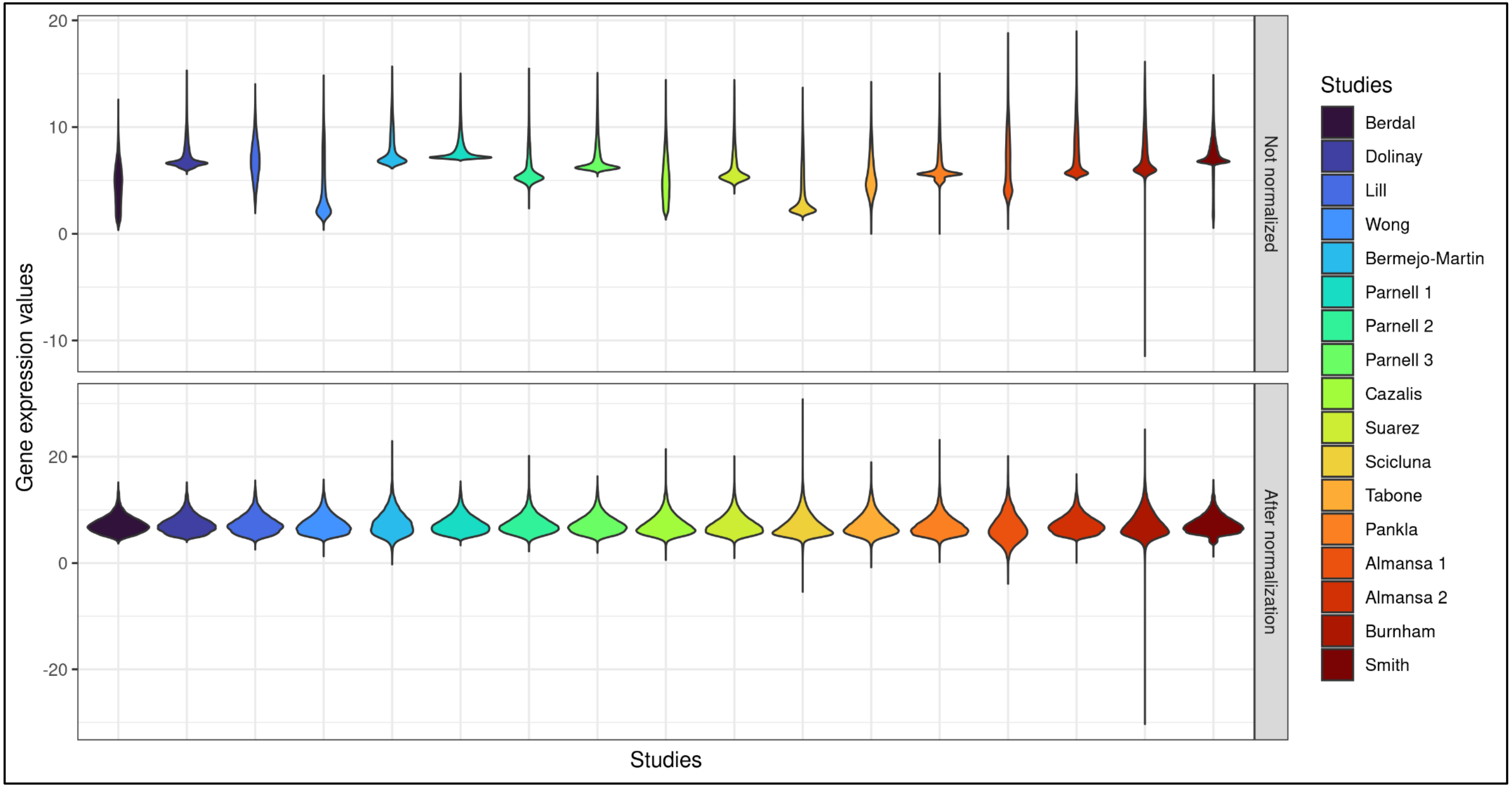

Supplementary figure 2. Effect of ComBat co-normalization at the gene level (here CD3D) in controls and cases in the 17 microarray studies

These density plots were computed with gene expression of CD3D in each study, in controls (n=517, left panel) and sepsis patients (n=1953, right panel), before and after (grey-dashed curves) co-normalization.

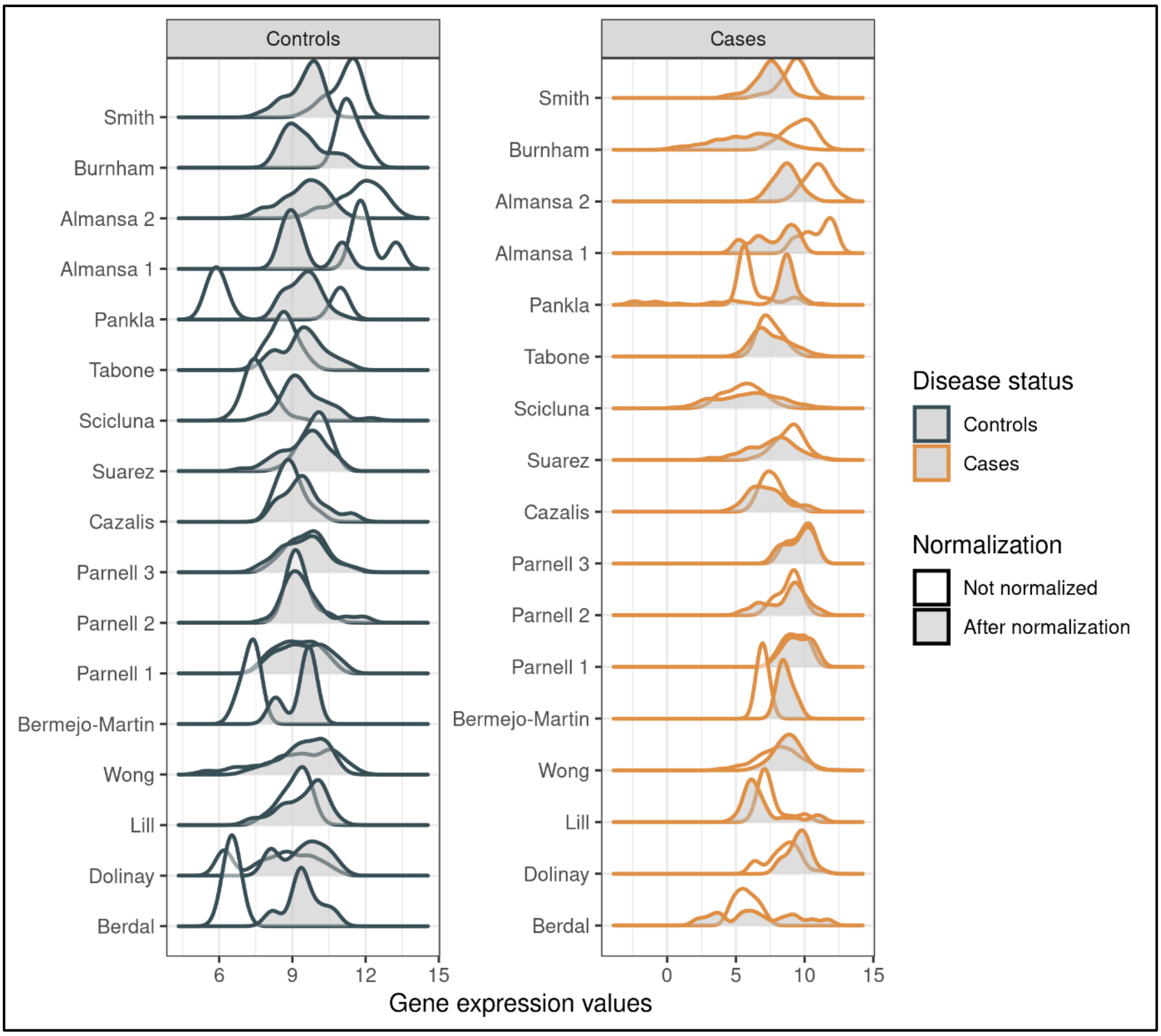

Supplementary figure 3. Effect of ComBat co-normalization using controls on gene expression levels for CEACAM1 (linked to sepsis) and CLDN8 (housekeeping gene) in the 17 microarray studies

This plot compares the effect of co-normalization on genes with minimal (here CLDN8) and high (here CEACAM1) differential expression between cases and controls. It shows the effect of co-normalization: 1) in control patients, where GE of both CLDN8 and CEACAM1 have similar means and variances across all studies, and 2) in cases where similar linear transformation enables to pool GE across studies for comparison of alive and deceased patients.

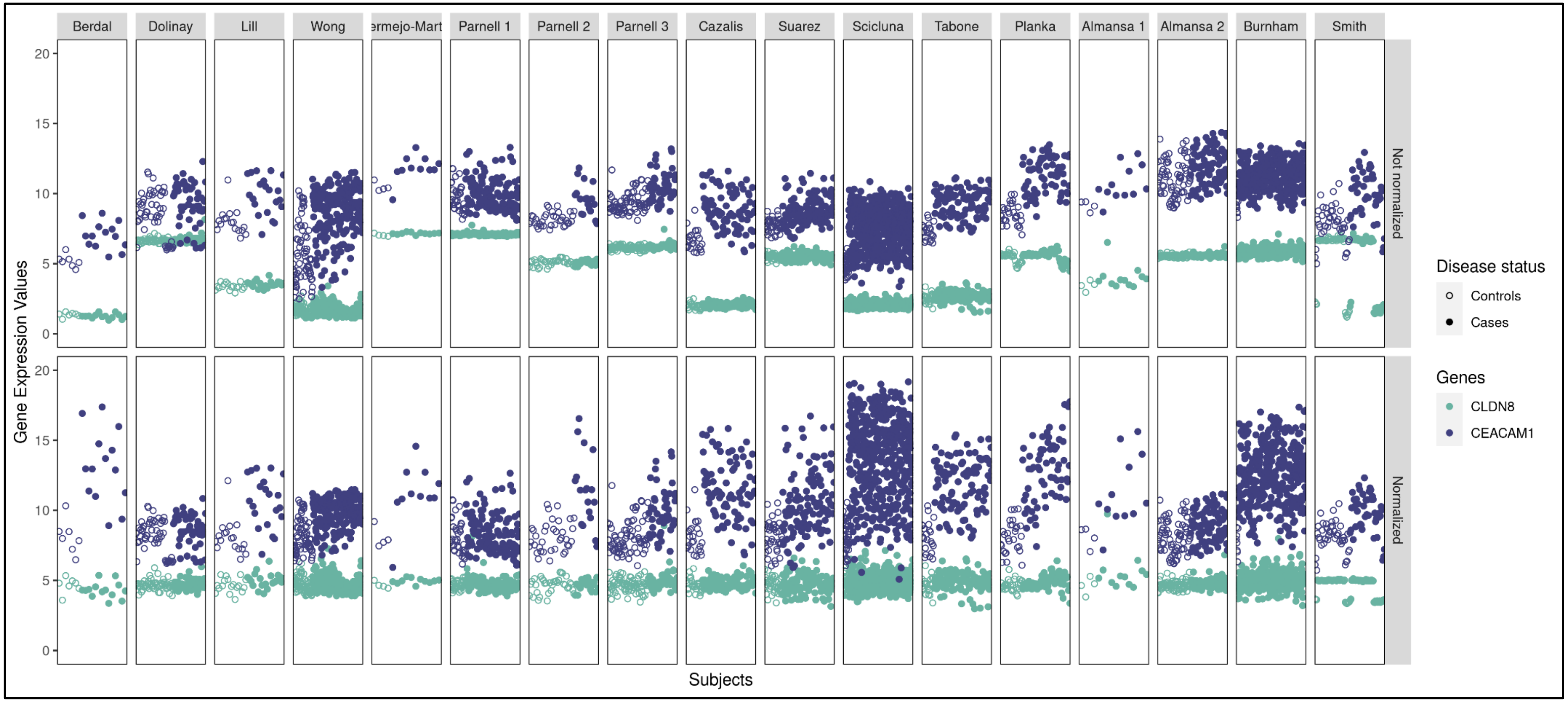

Supplement: Supplementary file 1 [file Data_Sheet_1.pdf]
